# *MORC2* gene de novo mutation leads to Charcot–Marie–Tooth disease type 2Z: A pediatric case report and literature review

**DOI:** 10.1097/MD.0000000000027208

**Published:** 2021-09-17

**Authors:** Haiyan Yang, Sai Yang, Qingyun Kang, Liming Yang, Hongmei Liao, Liwen Wu

**Affiliations:** Department of Neurology, Hunan Children's Hospital, P.R. China.

**Keywords:** Charcot–Marie–Tooth disease type 2Z, Leigh syndrome, *MORC2* gene

## Abstract

**Rationale:**

Mutations of the *MORC2* gene have most commonly been associated with autosomal-dominant Charcot–Marie–Tooth disease type 2Z (CMT 2Z), while the impact of *MORC2* mutations in CMT 2Z on neuronal biology and their phenotypic consequences in patients remain to be clarified.

**Patient concerns:**

We reported a 27-month-old child with a developmental lag of more than 1 year. He had progressive fatigue for 4 months, accompanied by dysphagia, choking while eating, and progressive aggravation. A genetic study revealed a de novo variant of *MORC2*, which has not yet been reported.

**Diagnosis:**

According to the child's clinical manifestations, genetic pattern, and American College of Medical Genetics and Genomics pathogenicity analysis, the patient was diagnosed with CMT 2Z caused by *MORC2* gene mutation.

**Interventions:**

Mitochondrial cocktail therapy (arginine, vitamin B1 tablets, vitamin B2 tablets, coenzyme Q10 capsules, L-carnitine oral liquid, idebenone tablets, etc) was given.

**Outcomes:**

Mitochondrial cocktail therapy did not significantly improve the child's condition, head magnetic resonance imaging lesions were not significantly improved at outpatient follow-up more than 1 month later, and the lesions were basically unchanged.

**Lessons:**

The clinical manifestations of the disease were similar to those of Leigh syndrome, and they were not significantly improved by cocktail therapy. This site has not been reported in the literature domestically or abroad, and the pathogenesis of CMT 2Z caused by this site mutation is indeed not related to mitochondrial dysfunction. Our study is helpful for clinicians with regard to the differential diagnosis of Leigh syndrome and CMT 2Z and improvement of clinicians’ understanding of CMT 2Z disease.

## 1 Introduction

The *MORC2* gene encodes a DNA-dependent ATPase that is a member of a family of ATPases fundamental for epigenetic silencing through chromatin modification. This gene plays a role in chromatin remodeling, DNA repair, and transcriptional regulation.^[1]^ It has most commonly been associated with autosomal-dominant Charcot–Marie–Tooth disease type 2Z (CMT 2Z), a form of axonal neuropathy with progressive weakness, muscle cramps, and sensory impairment presenting in childhood or early adulthood. However, there have been reports of some individuals presenting with hypotonia, generalized muscle weakness, and delayed milestones or occasionally with spinal muscular atrophy, intellectual disability, hearing loss, pyramidal signs, microcephaly, and brain atrophy in infancy.^[1]^ While genetic data have clearly established the causative role of *MORC2* in CMT 2Z, the impact of its mutations on neuronal biology and their phenotypic consequences in patients remain to be clarified.^[2]^ Sanchez-Solana et al^[3]^ found that cytosolic *MORC2* played a role in lipogenesis, adipogenic differentiation, and lipid homeostasis. The clinical manifestations of CMT 2Z caused by *MORC2* gene mutation are similar to those of Leigh syndrome, and whether *MORC2* gene mutation disrupts mitochondrial function has not yet been studied.^[1]^ We report one such case from a family and the results of investigating mitochondrial function in peripheral blood.

## 2 Methods

The patient was managed at the Department of Neurology, Hunan Children's Hospital. The parents of the patient provided written, informed consent. This study was approved by the Medical Ethics Committee of Hunan Children's Hospital.

### 2.1 Whole-exome sequencing of peripheral blood

The whole-exome sequencing method was performed according to our previous research methods.^[4]^ Whole-exome sequencing was performed simultaneously on samples from the patient, his father and his mother.

### 2.2 Full-length sequencing of the mitochondrial genome

Full-length sequencing of the mitochondrial genome of the child's peripheral blood, urine and oral mucosa cells was performed according to our previous research methods.^[4]^

### 2.3 Mitochondrial function test

We measured the mitochondrial membrane potential (ΔΨm) using peripheral blood samples to evaluate mitochondrial function. Peripheral blood leukocytes treated with JC-1 for 15 minutes were also analyzed by flow cytometry.

## 3 Case report

### 3.1 Clinical information

**Figure 1:**
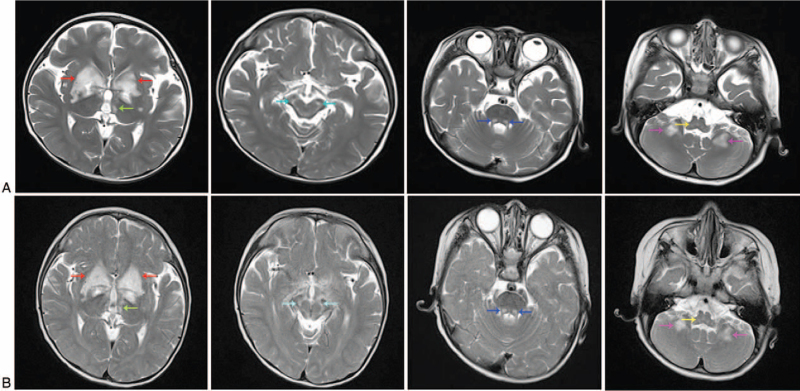
A. Head MRI showing the lesions of the child before treatment. B. Head MRI showing lesions more than 1 month after mitochondrial cocktail treatment. Red arrow: bilateral basal ganglia; green arrow: thalamus; blue arrow: cerebral peduncle; purple arrow: pons; yellow arrow: medulla oblongata; magenta arrow: dentate nucleus. There was no significant change in the head MRI findings after mitochondrial cocktail treatment. MRI = magnetic resonance imaging.

The child was 27 months old and had a developmental lag of more than 1 year. He had progressive fatigue for 4 months, accompanied by dysphagia, choking on eating, and progressive aggravation. Laboratory examination showed an elevated lactic acid level (5.04 mmol/L). Head magnetic resonance imaging (MRI) showed symmetrical patchy long T1 and long T2 signal shadows at the bilateral basal ganglia, thalamus, cerebral peduncle, pons, medulla oblongata, and dentate nucleus (Fig. 1A). Magnetic resonance spectroscopy showed obviously inverted bimodal lactate peaks. The electrophysiological examination of motor and sensory nerves showed that the nerve compound muscle action potential amplitude was decreased, while the conduction velocity and latency were basically normal, as detailed in Table 1. According to the child's clinical manifestations, laboratory results and head imaging findings, a comprehensive analysis of suspected Leigh syndrome was considered, and mitochondrial cocktail therapy (arginine, vitamin B1 tablets, vitamin B2 tablets, coenzyme Q10 capsules, L-carnitine oral liquid, idebenone tablets, etc) was given. After 5 days of treatment with mitochondrial cocktail therapy, the condition of the child was not significantly improved. After the child was discharged from the hospital, mitochondrial cocktail therapy was continued; nevertheless, the head MRI lesions showed no significant improvement at outpatient follow-up more than 1 month later and appeared essentially unchanged (Fig. 1B).

**Table 1:** Electrophysiological results of the patient's motor and sensory nerves.

Motor nerves	Lat SD (ms)	Amp SD (mV)	CV SD (m/s)
Right medianus	2.2 (2.31 ± 0.31)	2.8 (6.97 ± 2.28)	45.7 (49.43 ± 4.52)
Left medianus	2.8 (2.31 ± 0.31)	2.3 (6.97 ± 2.28)	47.7 (49.43 ± 4.52)
Left ulnaris	1.75 (1.85 ± 0.24)	3.3 (8.06 ± 1.48)	46.9 (51.40 ± 4.83)
Right tibialis	2.8 (2.37 ± 0.39)	6.2 (16.48 ± 3.18)	44.4 (43.79 ± 2.04)
Left tibialis	2.7 (2.37 ± 0.39)	4.6 (16.48 ± 3.18)	41.2 (43.79 ± 2.04)
Right peroneus	3.0 (2.37 ± 0.39)	0.9 (16.48 ± 3.18)	42.1 (48.35 ± 4.51)
Left peroneus	3.0 (2.37 ± 0.39)	0.6 (16.48 ± 3.18)	45.4 (48.35 ± 4.51)
Sensory nerves	Lat SD (ms)	Amp SD (uV)	CV SD (m/s)
Right medianus	1.40 (1.78 ± 0.20)	13 (17.60 ± 3.32)	44.3 (52.11 ± 4.03)
Left medianus	1.33 (1.78 ± 0.20)	13 (17.60 ± 3.32)	48.9 (52.11 ± 4.03)
Right ulnaris	1.17 (1.70 ± 0.15)	8.7 (16.62 ± 3.47)	44.4 (51.40 ± 4.19)
Left ulnaris	1.15 (1.70 ± 0.15)	8.8 (16.62 ± 3.47)	45.2 (51.40 ± 4.19)
Right peroneus super	1.85 (1.55 ± 0.20)	2.7 (19.34 ± 5.06)	54.1 (46.16 ± 4.11)
Left peroneus super	1.85 (1.55 ± 0.20)	2.4 (19.34 ± 5.06)	51.4 (46.16 ± 4.11)

CV = conduction velocity.

### 3.2 Genetic evaluation

**Figure 2:**
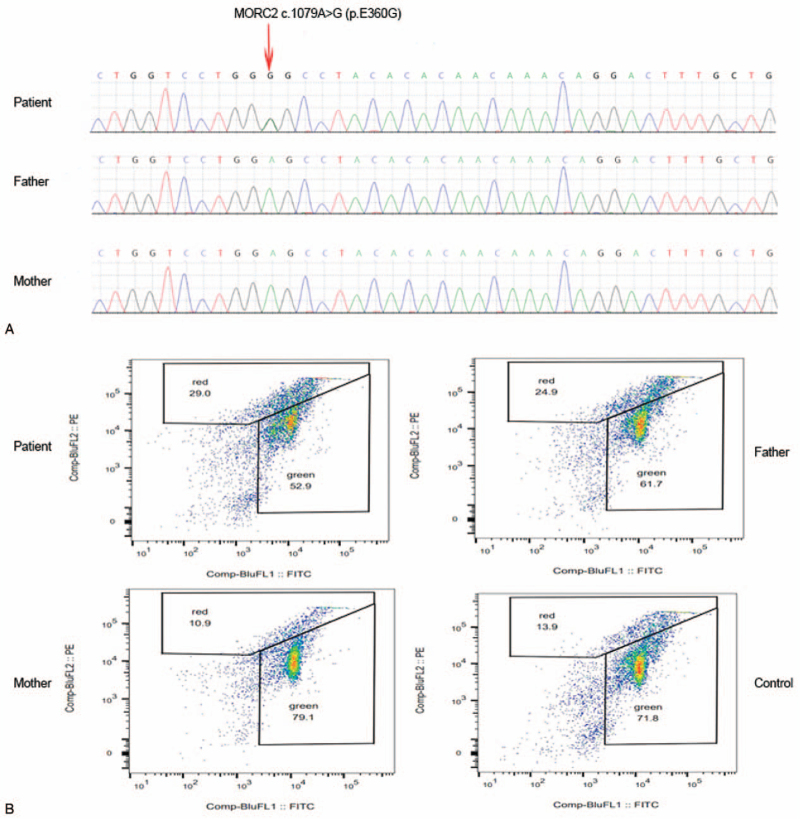
A. The results of whole-exome sequencing in patients with Charcot–Marie–Tooth disease type 2Z. There was a c.1079A>G (p.E360G) mutation in the *MORC2* gene of the child. The child's father and mother did not carry the mutation. B. Mitochondrial membrane potential. Flow cytometric analysis of fresh peripheral blood leukocytes in the patient, his parents and controls stained with JC-1. The ratio of red to green fluorescence was not significantly different between the patient and controls.

Analysis of peripheral blood from the child revealed the *MORC2* gene heterozygous mutation c.1079A>G (p. E360G). However, the sequencing data showed that the parents did not carry the mutation; thus, it could be a de novo mutation (Fig. 2A). At the same time, full-length sequencing of the mitochondrial genome of the peripheral blood, urine and oral mucosa cells of the child did not reveal pathogenic mutations related to the clinical manifestations.

### 3.3 Mitochondrial function evaluation

Fresh peripheral blood leukocytes from the patient, his parents and controls were stained with JC-1 and analyzed by flow cytometry. The results revealed no significant difference in the ratio of red to green fluorescence between the patient and controls (Fig. 2B), indicating that there was no damage to the ΔΨm in the peripheral blood leukocytes of the patient. This finding confirmed that this site mutation was indeed not related to mitochondrial dysfunction.

## 4 Literature review

We used the *MORC2* gene as a key word to search the PubMed database for cases of *MORC2* gene mutations that have previously been reported and summarized the reported *MORC2* gene mutation sites and their corresponding clinical phenotypes. To date, *MORC2* gene mutations have been reported in 55 children (Table 2). Their clinical phenotypes include CMT 2Z, facial dysmorphism, precocious puberty, strabismus, frequent respiratory infections, hirsutism, cataracts, hearing loss, hammertoes, delayed puberty, growth hormone deficiency, hypothyroidism, vitamin D deficiency, mildly increased prolactin, pyramidal signs, and mental retardation.^[1,5–8]^ Of the mutational sites reported in the literature,^[1,5–8]^ the following de novo mutations affected 19 patients: c.71 C>T (p.T24I) (1), c.79G>A (p.G27L) (4), c.260C>T (p.S87L) (4), c.263 C>T (p.A88V) (1), c.394C>T (p.A132C) (3), c.798 G>C (p.A266S) (1), c.1164C>G (p.S388A) (1), c.1181A>G (p.T394C) (2), c.707A>G (p.E236G) (1), and c.754C>T (p.R252W) (1). Eight individuals had maternal mutations, namely, c.1237G>T (p.V413P) (1) and c.1210G>A (p.A404A) (7). Additionally, 28 children carried unknown mutations: c.79G>A (p.G27L) (1), c.260C>T (p.S87L) (1), c.394C>T (p.A132C) (1), c.395 G>T (p.A132L) (1), c.1164C>G (p.S388A) (1), c.1181A>G (p.T394C) (1), c.1237G>T (p.V413P) (1), c.707A>G (p.E236G) (1), c.848G>A (p.R283H) (1), c.1753C>T (p.R585C) (1), c.1396G>C (p.D456H) (1), c.2270A>G (p.E757G) (1), c.743A>G (p.Y248C) (1), c.1330C>T (p.G444R) (1), c.286C>G (p.Q96E) (1), c.754C>T (p.A190T) (8), c.1013A>G (p.G338>A) (1), c.995A>G (p.T332C) (1), c.1034G>A (p.C345T) (1), and c.1106C>T (p.A369V) (2). A diagnosis of mitochondrial disease was suspected in 5 individuals due to their Leigh-like lesions on cranial MRI.^[1]^ The mutations sites in these 5 individuals were c.71 C>T (p.T24I) (1), c.79G>A (p.G27L) (2), c.260C>T (p.S87L) (1), and c.394C>T (p.A132C) (1).

**Table 2:** Summary of the reported *MORC2* reported cases.

Reference	Mutation	Case (n)	Inheritance (n)	Sex (n)	Age (m)	Brain MRI (n)	EMG (n)	Clinical feature
Guillen Sacoto MJ et al (2020)	c.71 C>T (p.T24I)	1	De novo (1)	F (1)	4.2 y	Leigh syndrome-like lesions (1);	N/A	CMT 2Z; facial dysmorphism; precocious puberty; strabismus
Guillen Sacoto MJ et al (2020)	c.79G>A (p.G27L)	5	De novo (4); unknown (1)	M (2); F (3)	4.8 y	Leigh syndrome-like lesions (2); N (3)	N/A (4); N (1);	CMT 2Z; hearing loss; delayed puberty, growth hormone deficiency, hypothyroidism; vitamin D deficiency; precocious puberty, mildly increased prolactin
Guillen Sacoto MJ et al (2020)	c.260C>T (p.S87L)	5	De novo (4); unknown (1)	M (3); F (2)	5.0 y	Leigh syndrome-like lesions (1); A (1); N (3)	A(5)	CMT 2Z; frequent respiratory infections; hirsutism; Cataract
Guillen Sacoto MJ et al (2020)	c.263 C>T (p.A88V)	1	De novo (1)	F (1)	23.0 y	N/A	N/A	CMT 2Z; hearing loss; hammertoes
Guillen Sacoto MJ et al (2020)	c.394C>T (p.A132C)	4	De novo (3); unknown (1)	M (3); F (1)	11.7 y	Leigh syndrome-like lesions (1); A (2); N (1)	A (3); N/A (1)	CMT 2Z; facial dysmorphism; hearing loss; hammertoes
Guillen Sacoto MJ et al (2020)	c.395 G>T (p.A132L)	1	Unknown (1)	M (1)	30.0 y	N/A	A (1)	CMT 2Z; hearing loss
Guillen Sacoto MJ et al (2020)	c.798 G>C (p.A266S)	1	De novo (1)	F (1)	12.0 y	A (1)	N/A	CMT 2Z; facial dysmorphism; hearing loss
Guillen Sacoto MJ et al (2020)	c.1164C>G (p.S388A)	2	De novo (1); unknown (1)	F (2)	12.0 y	A (2)	N (1); N/A (1)	CMT 2Z; facial dysmorphism; hearing loss
Guillen Sacoto MJ et al (2020)	c.1181A>G (p.T394C)	3	De novo (2); unknown (1)	F (1); M (2)	29.0 y	A (2); N/A (1)	A (3)	CMT 2Z
Guillen Sacoto MJ et al (2020)	c.1237G>T (p.V413P)	2	Maternal (1); unknown (1)	F (2)	17.3 y	N (1); N/A (1)	N/A (2)	CMT 2Z; facial dysmorphism; hearing loss; growth hormone deficiency
Albulym OM, et al (2016)	c.707A>G (p.E236G)	2	De novo (1); unknown (1)	M (2)	N/A	N/A	N/A	CMT 2Z
Albulym OM, et al (2016)	c.754C>T (p.R252W)	1	De novo (1)	F (1)	N/A	N/A	A (1)	CMT 2Z and pyramidal signs
Albulym OM, et al (2016)	c.848G>A (p.R283H)	1	Unknown (1)	N/A	N/A	N/A	N/A	CMT 2Z
Albulym OM, et al (2016)	c.1753C>T (p.R585C)	1	Unknown (1)	N/A	N/A	N/A	N/A	CMT 2Z
Albulym OM, et al (2016)	c.1396G>C (p.D456H)	1	Unknown (1)	N/A	N/A	N/A	N/A	CMT 2Z
Albulym OM, et al (2016)	c.2270A>G (p.E757G)	1	Unknown (1)	N/A	N/A	N/A	N/A	CMT 2Z
Albulym OM, et al (2016)	c.743A>G (p.Y248C)	1	Unknown (1)	N/A	N/A	N/A	N/A	CMT 2Z
Albulym OM, et al (2016)	c.1330C>T (p.G444R)	1	Unknown (1)	N/A	N/A	N/A	N/A	CMT 2Z
Albulym OM, et al (2016)	c.286C>G (p.Q96E)	1	Unknown (1)	N/A	N/A	N/A	N/A	CMT 2Z
Ando M, et al (2017)	c.754C>T (p.A190T)	8	Unknown (8)	F (2); M (6)	30.0 y	N (8)	A (8)	CMT 2Z and mental retardation
Ando M, et al (2017)	c.1013A>G (p.G338>A)	1	Unknown (1)	M (1)	44.0 y	N (1)	N/A	CMT 2Z
Ando M, et al (2017)	c.995A>G (p.T332C)	1	Unknown (1)	M (1)	29.0 y	N (1)	A (1)	CMT 2Z
Ando M, et al (2017)	c.1034G>A (p.C345T)	1	Unknown (1)	M (1)	15.0 y	N (1)	A (1)	CMT 2Z
Ando M, et al (2017)	c.1106C>T (p.A369V)	2	Unknown (2)	M (2)	25.5 y	A (1); N (1)	A (2)	CMT 2Z and mental retardation
Semplicini C, et al (2017)	c.1210G>A (p.A404A)	7	Maternal (7)	F (3); M (4)	36.5 y	N (7)	A (7)	CMT 2Z

A = abnormal, CMAP = compound muscle action potential (mV), CMT 2Z = Charcot–Marie–Tooth disease type 2Z, F = female, M = male, m = medium, MRI = magnetic resonance imaging, N = normal, n = number, N/A = not available, y = year.

## 5 Discussion

The association of *MORC2* variants with human disease has been noted only recently, and most of the affected individuals have been identified through studies of neuropathies.^[1]^*MORC2* mutations are responsible for axonal motor and sensory neuropathies with a congenital or infantile onset and a presentation similar to that of spinal muscular atrophy. Symptoms in childhood or juvenile onset start distally and progress to involve proximal muscles in an asymmetrical and random fashion, causing severe disability in adults. Significant positive motor activity, such as cramps, fasciculations, and myokymia, is present from the beginning.^[5]^ Currently, the clinical phenotypes reported in children with *MORC2* gene mutations include CMT 2Z, facial dysmorphism, precocious puberty, strabismus, frequent respiratory infections, hirsutism, cataracts, hearing loss, hammertoes, delayed puberty, growth hormone deficiency, hypothyroidism, vitamin D deficiency, mild prolactin elevation, and pyramidal signs.^[5–8]^ A total of 19 de novo mutations have been reported in the literature.^[5–8]^ Five individuals for whom brain imaging data were available had lesions similar to those observed in Leigh syndrome.^[1]^ CMT 2Z is an autosomal-dominant disease caused by *MORC2* gene mutation and clinically manifests as muscle spasms caused by peripheral neuropathy, distal muscle weakness, and muscle atrophy, hypotonia, distal sensory disturbance, etc. Usually, CMT 2Z onset occurs at approximately 10 to 20 years of age, while some patients exhibit hypotonia, muscle weakness, and developmental delay after birth or in infancy.^[7,8]^

Herein, we report the case of a patient with clinical manifestations similar to those of Leigh syndrome and a de novo *MORC2* gene mutation, c.1079A>G (p.E360G), that was not carried by the parents. The *MORC2* c.1079A>G (p.E360G) variant has not previously been reported in the related literature. To date, this mutation has not been reported in our reference population gene database, and according to the American College of Medical Genetics and Genomics variation classification guidelines, this variation is class 2 likely pathogenic. Therefore, on the basis of the combination of the child's clinical manifestations, genetic pattern and the result of the American College of Medical Genetics and Genomics pathogenicity analysis, the patient was diagnosed with CMT 2Z caused by *MORC2* gene mutation. In addition, considering that the clinical presentation of the child was similar to that of Leigh syndrome and that mitochondrial cocktail therapy did not significantly improve the child's condition, we conducted an in-depth functional study of the pathogenic mechanism of this gene locus and found that there was no significant difference in the ΔΨm among the child, his parents and healthy controls, suggesting that the pathogenic mechanism of this gene mutation is indeed not related to mitochondrial dysfunction.

In conclusion, we reported a case of the de novo *MORC2* gene mutation c.1079A>G (p.E360G) in a child with CMT 2Z. The clinical manifestations of the disease were similar to those of Leigh syndrome, and cocktail therapy did not lead to significant improvements. This site has not previously been reported in the domestic or foreign literature, and the pathogenesis of CMT 2Z caused by this site mutation is indeed not related to mitochondrial dysfunction. These findings will be helpful for clinicians with regard to the differential diagnosis of Leigh syndrome and CMT 2Z and improvement of clinicians’ understanding of CMT 2Z.

## Acknowledgments

We wish to thank the patient's family for participation in this study.

## Author contributions

HY conducted the literature review and drafted the manuscript. SY, QK, LY, and HL made substantial contributions to the conception and interpretation of data. LW was responsible for revising the manuscript critically and gave final approval of the version to be published. All authors read and approved the manuscript.

**Data curation:** Sai Yang.

**Formal analysis:** Liming Yang.

**Funding acquisition:** Hongmei Liao.

**Resources:** Qingyun Kang.

**Writing – original draft:** Haiyan Yang.

**Writing – review & editing:** Liwen Wu.
